# Unity Makes Strength: Exploring Intraspecies and Interspecies Toxin Synergism between Phospholipases A_2_ and Cytotoxins

**DOI:** 10.3389/fphar.2020.00611

**Published:** 2020-05-07

**Authors:** Manuela B. Pucca, Shirin Ahmadi, Felipe A. Cerni, Line Ledsgaard, Christoffer V. Sørensen, Farrell T. S. McGeoghan, Trenton Stewart, Erwin Schoof, Bruno Lomonte, Ulrich auf dem Keller, Eliane C. Arantes, Figen Çalışkan, Andreas H. Laustsen

**Affiliations:** ^1^ Medical School, Federal University of Roraima, Boa Vista, Brazil; ^2^ Department of Biotechnology and Biomedicine, Technical University of Denmark, Kongens Lyngby, Denmark; ^3^ Department of Biotechnology and Biosafety, Graduate School of Natural and Applied Sciences, Eskişehir Osmangazi University, Eskişehir, Turkey; ^4^ Department of BioMolecular Sciences, School of Pharmaceutical Sciences of Ribeirão Preto, University of São Paulo, Ribeirão Preto, Brazil; ^5^ Department of Biology, Lund University, Lund, Sweden; ^6^ Facultad de Microbiología, Instituto Clodomiro Picado, Universidad de Costa Rica, San José, Costa Rica; ^7^ Department of Biology, Faculty of Science and Art, Eskişehir Osmangazi University, Eskişehir, Turkey

**Keywords:** toxin synergism, phospholipase A_2_, toxin complexes, cytotoxins, melittin, cytotoxicity, toxin interactions, venom

## Abstract

Toxin synergism is a complex biochemical phenomenon, where different animal venom proteins interact either directly or indirectly to potentiate toxicity to a level that is above the sum of the toxicities of the individual toxins. This provides the animals possessing venoms with synergistically enhanced toxicity with a metabolic advantage, since less venom is needed to inflict potent toxic effects in prey and predators. Among the toxins that are known for interacting synergistically are cytotoxins from snake venoms, phospholipases A_2_ from snake and bee venoms, and melittin from bee venom. These toxins may derive a synergistically enhanced toxicity *via* formation of toxin complexes by hetero-oligomerization. Using a human keratinocyte assay mimicking human epidermis *in vitro*, we demonstrate and quantify the level of synergistically enhanced toxicity for 12 cytotoxin/melittin-PLA_2_ combinations using toxins from elapids, vipers, and bees. Moreover, by utilizing an interaction-based assay and by including a wealth of information obtained *via* a thorough literature review, we speculate and propose a mechanistic model for how toxin synergism in relation to cytotoxicity may be mediated by cytotoxin/melittin and PLA_2_ complex formation.

## Introduction

The venomous animals that pose a threat to human health are classified in six major groups: cnidarians, venomous fish, scorpions, spiders, hymenopterans, and snakes (Ericsson et al., 2006). Their venoms are complex cocktails of toxic proteins, peptides, and small organic and inorganic molecules. In general, venoms derive their toxicity from proteins known as toxins. These toxins are in themselves a diverse and complex group, including smaller neurotoxic peptides, larger phospholipases, and venom proteases, along with many other protein families (Ducancel, 2016). In fact, it is estimated that between 19,000 and 25,000 snake toxins, 100,000 scorpion toxins, more than 10 million spider toxins, and a large unknown number of toxins from other venomous creatures exist (Laustsen et al., 2016a; Laustsen et al., 2016b).

Venoms are produced for defensive and/or predatory purposes to provide a survival benefit to the species possessing them. However, the production and replenishment of these venoms come with a metabolic cost for the venomous animals (Morgenstern and King, 2013). This metabolic cost has forced venomous animals to evolve mechanisms for minimizing venom expenditure, such as the venom optimization hypothesis (Wigger et al., 2002) and toxin synergism (Laustsen, 2016). Indeed, snakes (Strydom, 1976; Mora-Obando et al., 2014; Lauridsen et al., 2016), spiders (Chan et al., 1975; Wullschleger, 2005), scorpions (Lazarovici et al., 1984), and bees (Mingarro et al., 1995) have evolved to produce venoms with potencies that are larger than the sum of the individual toxins (toxin synergism). One of the most prominent examples of this phenomenon is the synergy between cytotoxins and phospholipases, which was reported for the first time more than half a century ago (Condrea et al., 1964). Cytotoxins from snakes belong to the three-finger toxin (3FTx) superfamily of proteins and share a common scaffold of three loops of β-strands extending from a central globular core reticulated by four highly conserved disulphide bridges (Kessler et al., 2017). Researchers have used different names for categorizing these toxins, including membrane-active polypeptides, membrane-disruptive polypeptides, membrane toxins, membranotoxins, cardiotoxins (Harvey, 2018), direct lytic factors (DLF) (Slotta and Vick, 1969), and cobramines (Wolff et al., 1968). However, in 1988, Dufton and Hider adopted the name “cytotoxin,” which underlines the fact that this group of toxins can kill different cell types by interacting with and disrupting their membranes (Dufton and Hider, 1988). The term “cytotoxin” has since then been more widely adopted in the literature and will be the term used in this study. Melittin, the main toxic component of bee venoms, was also first identified as a DLF, and as its mechanism closely resembles that of cytotoxins (Dempsey, 1990), it may be considered as a “cytotoxin-like” peptide.

While snake cytotoxins are mainly found in the genera *Naja* and *Hemachatus* (Dufton and Hider, 1988), snake venom phospholipases A_2_ (svPLA_2_s) are found in all venomous snake families (i.e., Viperidae, Elapidae, Atractaspididae, and Colubridae) (Kini, 1997; Xiao et al., 2017). Catalytically active svPLA_2_s hydrolyze membrane glycerophospholipids at the *sn*-2 site of these molecules (Xiao et al., 2017), however, many svPLA_2_s have over the course of evolution lost their catalytic activity, yet retain toxicity via other functions, such as an ability to disrupt cellular membranes very selectively (Arni and Ward, 1996; Kini, 2003). Based on their molecular structure, svPLA_2_s can be classified into three groups. 1) Group IA contains seven disulfide bridges and a characteristic surface loop between residues 63 and 67, called the elapidic loop. This group is primarily found in Elapidae, although some have also been reported for Colubridae. 2) Group IIA contains a seven-residue C-terminal extension and seven conserved disulfide bonds and is found in Viperidae. 3) Group IIB has a six-residue C-terminal extension and only six disulfide bridges, which means it lacks an otherwise universally conserved 61–95 disulfide bond. This group of svPLA_2_s is found in vipers (Six and Dennis, 2000). A different group of phospholipases A_2_, group III, can be found in lizard and bee venoms. Group III PLA_2_s have molecular masses that are higher than the molecular masses of PLA_2_s from snakes (15–18 kDa compared to 13–15 kDa, respectively) and contain eight disulfide bridges (Dennis et al., 2011).

The first synergistic effect between PLA_2_s and cytotoxins was reported by Condrea and Mager in 1964. Their study (Condrea et al., 1964) demonstrated, using erythrocytes, that when lower concentrations of *Naja naja* PLA_2_ causing no significant hemolysis or phospholipid hydrolysis (3.3% hemolysis, 0% phospholipid hydrolysis) were combined with a cobra cytotoxin with no phospholipase activity, significant hemolysis and phospholipid hydrolysis was observed (86.5% hemolysis, 77% phospholipid hydrolysis) (Condrea et al., 1964). In a later study (Klibansky et al., 1968), it was shown that synergism also occurred between PLA_2_s from *Vipera palestinae* and cytotoxins from cobras, implying that synergism was not restricted to toxins from the same animal. In 1995, the occurrence of synergism between cytotoxin P4 from *N. nigricollis* and homologous PLA_2_s along with many heterologous PLA_2_s was investigated using melanoma tumor cells. Here, sublytic concentrations of cytotoxin P4 combined with non-lytic concentrations of PLA_2_s from *N. nigricollis, N. atra, N. melanoleuca, Walterinnesia aegyptia, Bitis arietans*, and *Pseudocerastes persicus,* along with porcine pancreas and bee venom, were demonstrated to cause 100% cell lysis (Chaim-Matyas et al., 1995). For bees, similar examples have been reported, where PLA_2_ from bee venom (bvPLA_2_) has been shown to synergistically increase the lytic effect of melittin (Yunes et al., 1977; Frangieh et al., 2019; Pucca et al., 2019).

Despite the considerable amount of research that has been performed in the field of toxinology, little is still known about the phenomenon of toxin synergism. In particular, the synergistic effects between cytotoxins and PLA_2_s, as well as other toxin-toxin combinations, remain understudied, and the molecular mode of interaction between many of these toxins, as well as their combined mechanism of action, are yet to be completely elucidated. In this study, we demonstrate how several different combinations between all three PLA_2_ groups (I, II, and III) and different cytotoxins from *N. nigricollis*, *N. mossambica*, *N. melanoleuca*, as well as melittin from bee venom interact synergistically using a cytotoxicity assay involving immortalized human keratinocytes. Based on the synergistically enhanced cytotoxic effects observed on the keratinocytes, an interaction-based assay, and a thorough literature review, this study also proposes a mechanistic model for how cell lysis is synergistically enhanced by cytotoxin and PLA_2_ complex formation.

## Materials and Methods

### Toxins

Venoms of *N. nigricollis* (the black-necked spitting cobra), *N. melanoleuca* (the forest cobra), and *N. mossambica* (the Mozambique spitting cobra) were purchased from Latoxan (Valence, France). Melittin (P01501) and bvPLA_2_ (P00630) were purchased from Sigma-Aldrich (Cotia, SP, Brazil, and St Louis, MO, USA, respectively). *Bothrops asper* myotoxin II (MII, lacking PLA_2_ activity, P24605) was isolated as previously described (Lomonte and Gutiérrez, 1989). Venoms of *N. nigricollis, N. melanoleuca*, and *N. mossambica* were fractionated by reversed-phase high-performance liquid chromatography (RP-HPLC) as described elsewhere (Lauridsen et al., 2017), and peaks were numbered according to (Petras et al., 2011; Lauridsen et al., 2017; Dehli, 2018), respectively. In order to evaluate toxin purities, the fractionated toxins were sent to the Proteomics Core at the Technical University of Denmark where *De Novo* sequencing was performed. The subsequent peptide spectra were screened against the Uniprot database using *N. naja* or *B. asper* as the identifier species. The snake cytotoxins were screened against *N. naja* and MII was screened against *Bothrops asper*. The cytotoxin and phospholipase A_2_ purities are shown in **Supplementary Table 1** and **Supplementary Figure 1**.

### Cell Culture and Synergy Assessment

An immortalized human keratinocyte cell line (N/TERT) (kindly provided by Edel O’Toole from the Queen Mary University of London) (Dickson et al., 2000) was cultured in Dulbecco’s modified Eagle’s medium (DMEM:F12; Grand Island, NY, USA) supplemented with 10% (v/v) fetal bovine serum (FBS), 1% (v/v) penicillin-streptomycin (Sigma, St. Louis, MO, USA), and 1 × RMplus supplement (McGeoghan, 2017), under standard conditions (37°C, 5% CO_2_, and 85% humidity). Sub-culturing was performed by incubating with 0.05% Trypsin-EDTA (Life technologies, Grand Island, NY, USA) for 5 to 10 min at 37°C to detach adherent cells. The cell suspension was diluted 1:1 with medium to neutralize the trypsin, and then centrifuged at 1,300*g* for 5 min. Approximately 4×10^3^ cells diluted in 100 µl of medium were seeded per well in 96-well polystyrene black opaque-plates (Thermo Fisher Scientific, Roskilde, DK) and incubated overnight under the standard conditions. The medium was aspirated and replaced by media (100 µl per well) containing different combinations of PLA_2_s (bvPLA_2_, MII, or Nmo12) and cytotoxins (fraction 18 from *N. nigricollis* (Nn18), fraction 20 from *N. nigricollis* (Nn20), fraction 17 from *N. melanoleuca* (Nm17), fraction 9 from *N. mossambica* (Nmo9)) or melittin, which had been co-incubated for 30 min at 37°C before addition. Controls consisted of wells without cells, cells incubated without addition of toxins, and cells incubated with only individual toxins. The plates were incubated under the standard conditions for 24 h. Cytotoxicity was evaluated by the CellTiter-Glo luminescent cell viability assay (Promega, Madison, WI, USA) which uses adenosine triphosphate (ATP) levels to measure living cells (Riss et al., 2011). The manufacturer’s protocol was followed. Experiments were performed in triplicate with two technical replicates for each combination, and results were expressed as mean ± SD. Data were evaluated through an analysis of variance (ANOVA) test followed by a Bonferroni post-test, and a significance level of *p* < 0.05 was used for statistical testing. All statistical analyses were performed using GraphPad-Prism 6 software (GraphPad-Prism Software Inc., San Diego, CA, USA).

### Combination Index

The Coefficient of Drug Interaction or Combination of Drug Index (CDI) (Zhao et al., 2014), here named Combination of Toxin Index (CTI), was calculated by the equation CTI = (*E*)_1,2_/*E*_1_ × *E*_2_, where (*E*)_1,2_ is the measured effect of the combination effect; *E*_1_ and *E*_2_ are the individual effects of each toxin. Thus, CTI values of <1, =1, or >1 indicate that the toxin–toxin interactions are synergistic, additive, or antagonistic, respectively.

### Toxin Biotinylation and Protein-Complex Isolation

The toxins MII, Nm17, and melittin were biotinylated using PEG_4_-conjugated biotin (EZ-Link™ NHS-PEG_4_-Biotin, Thermo Fisher Scientific, Rockford, IL, USA) with 1:1.5 molar ratio (toxin/biotin), as described elsewhere (Laustsen et al., 2017). In order to evaluate if the synergistically-acting toxins interact with each other and generate complexes, a pull-down assay was performed. Different combinations of toxins were used: biotinylated MII (bio-MII) + Nm17, biotinylated Nm17 (bio-Nm17) + bvPLA_2_, and biotinylated melittin (bio-melittin) + bvPLA_2_. The mixtures containing 5 µg of each toxin diluted in phosphate-buffered saline (PBS) pH 7.2 (final volume, 100 µl) were co-incubated for 1 h at 37°C and transferred to 30 µl of Dynabeads M-280 Streptavidin (Invitrogen, Trondheim, Norway). Beads and mixtures were incubated for 30 min at room temperature and mixed gently each 10 mins. As control, the same combinations were used with non-biotinylated toxins. Magnetic separation was used to collect the beads. The beads were washed three times with 200 µl of PBS pH 7.2. For elution of the toxins, 30 µl of PBS pH 7.2 containing lithium dodecyl sulphate loading buffer (NuPAGE LDS, Thermo Fisher Scientific, Rockford, IL, USA), and 0.1 M of dithiothreitol (DTT, Thermo Fisher Scientific, Rockford, IL, USA) were added to the beads and incubated 10 min at 70°C, after which the toxins were recovered from the supernatant. The samples obtained from the pull-down assay were analyzed through electrophoresis for low molecular weight proteins according to the method of Schagger and von Jagow (Schägger and von Jagow, 1987). Samples were run on a 16% Tris-Tricine SDS-PAGE gel (Novex Tricine Gels, Invitrogen, Carlsbad, CA, USA) at 149 V and 150 mA. The gel was stained with silver (SilverXpress, Life Technologies, Carlsbad, CA, USA).

## Results

### Cytotoxic Effects of Cross-Species Synergistic Combinations of Toxins

**Figure 1:**
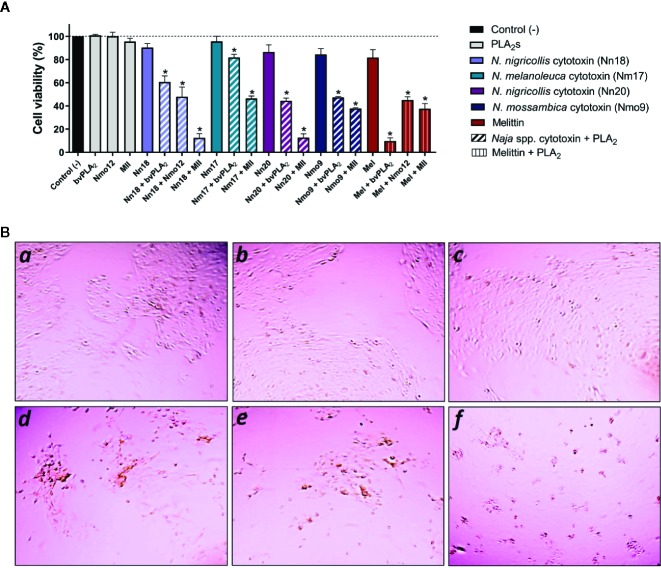
Synergistically enhanced lytic effects of combinations of cytotoxins and PLA_2_s from the same and different species. **(A)** Low lytic concentrations of cytotoxins (Nn18: 8 µg/ml, Nm17: 6 µg/ml, Nn20: 20 µg/ml, Nmo9: 1 µg/ml, and melittin: 5 µg/ml) were mixed with sublytic concentrations of PLA_2_s (Nmo12: 50 µg/ml, bvPLA_2_: 50 µg/ml, and MII: 12 µg/ml) and the combinations were added to human keratinocytes (N/TERT). Controls were performed with N/TERT cells not subjected to the toxins. Synergistically enhanced cytotoxicity was examined after 24 h of incubation by determination of adenosine triphosphate (ATP) levels through a luminescent cell viability assay. Experiments were performed in triplicates with two replicates for each combination, and results are expressed as mean ± SD. Data was analyzed by an analysis of variance (ANOVA) test followed by a Bonferroni post-test. (*p < 0.001 compared to the respective effect with the individual cytotoxin). **(B)** Representative morphological features of N/TERT cells captured by Evo XL imaging system using a 4× objective lens. (*a-c*) Standard N/TERT cell cultures forming islands of adherent and flattened keratinocytes, indicating viable cells: *a*: control; *b:* bvPLA_2_, and *c:* Nn18. (*d-f*) N/TERT cell cultures forming separated clusters of keratinocytes (fragmentation) and presenting several rounded cells, indicating cell damage and lysis: *d:* Nn18 + bvPLA_2_; *e:* Nn18 + Nmo12, and *f:* Nn18 + MII. Similar patterns were seen for other toxins.

Immortalized human keratinocytes (N/TERTs) were challenged with individual cytotoxins in different concentrations and analyzed for cell survival (data not shown). Based on these experiments, doses resulting in low cytotoxicity (0–20%), were used for challenging N/TERT cells. The isolated cytotoxins (Nn18, Nm17, Nn20, Nmo9) and melittin, as well as these cytotoxins in combination with three PLA_2_s (Nmo12, MII, and bvPLA_2_) from different species were added to the culture medium of the N/TERT cells and incubated for 24 h (**Figure 1**). Exposure of N/TERT cells to *Naja* spp. cytotoxins showed an average of seven-fold higher cytotoxic activity in combination with MII (~62%), five-fold higher in combination with Nmo12 (~50%), and four-fold higher when combined to bvPLA_2_ (~45%) (**Figure 1**). In contrast, melittin showed the highest cytotoxic effect in combination with bvPLA_2_, with five-fold higher cytotoxicity (~90%), and a three-fold higher cytotoxicity when combined with MII (~60%) and Nmo12 (~55%).

**Figure 2:**
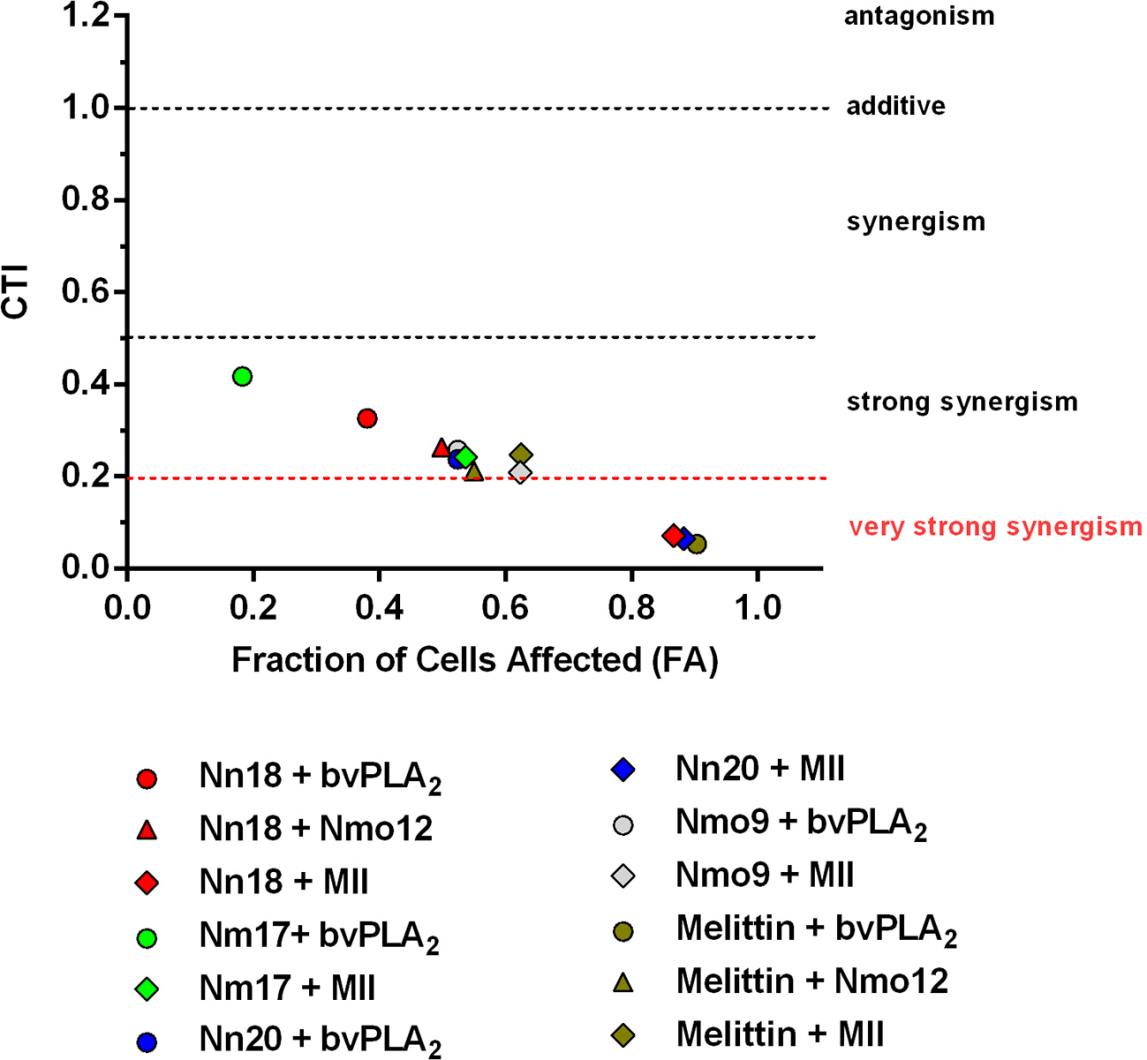
Combination effect of cytotoxins and PLA_2_s. The Combination of Toxin Index (CTI) was calculated for 12 different combinations of melittin/cytotoxins and PLA_2_s. The X axis shows the level of cytotoxicity, where 0 = 100% of viable cells, and 1 = 0% of viable cells. The Y axis shows the CTI values, where values <1, =1, or >1 indicate that the toxins act synergistically, additively, or antagonistically, respectively. CTI <0.5 indicates strong synergism, and CTI <0.2 very strong synergism. Triangles, diamonds, and circles represent melittin/cytotoxin combinations with PLA_2_s from group I (Nmo12), II (MII), and III (bvPLA_2_), respectively.

To provide evidence of the synergistically potentiated effects of the toxins in combination compared to the effects obtained by the single toxins, the CTI was calculated. All 12 cytotoxin/melittin-PLA_2_ combinations demonstrated a strong synergistic effect (CTI < 0.5). Moreover, three of the combinations (melittin + bvPLA_2_, Nn18 + MII, and Nn20 + MII) presented very strong synergism (CTI < 0.2) (**Figure 2**).

### Evidence of Formation of Phospholipase A_2_-Cytotoxin Complex

**Figure 3:**
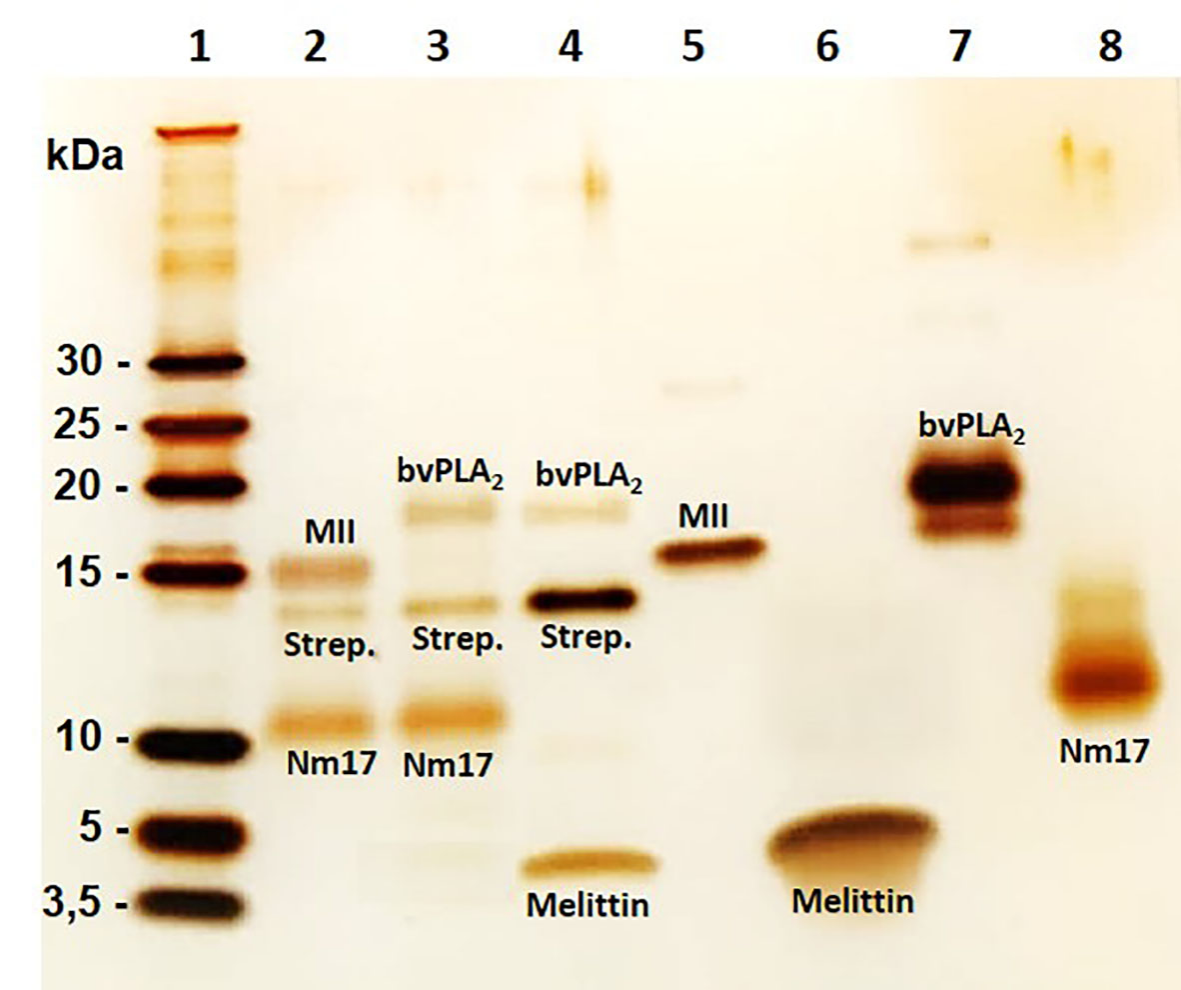
Electrophoresis profiles of toxins and toxin complexes isolated in a pull-down experiment. Different combinations of cytotoxins and PLA_2_s (5 µg of each and one of them biotinylated) were co-incubated for 1 h at 37°C. This was followed by a pull-down experiment using Dynabeads M280 Streptavidin. Toxins were eluted with lithium dodecyl sulphate (LDS) and dithiothreitol (DTT) and heated at 70°C for 10 min. Eluted toxins were evaluated using a Tris-Tricine SDS-PAGE 16% and silver staining. Lane 1: Ladder; Lane 2: bio-MII + Nm17; Lane 3: bio-Nm17 + bvPLA_2_; Lane 4: bio-melittin + bvPLA_2_; Lane 5: MII; Lane 6: melittin (control); Lane 7: bvPLA_2_ (control); Lane 8: Nm17 (control). No protein band was seen when combinations with non-biotinylated toxins were used (data not shown). Strep. means streptavidin.

**Figure 4:**
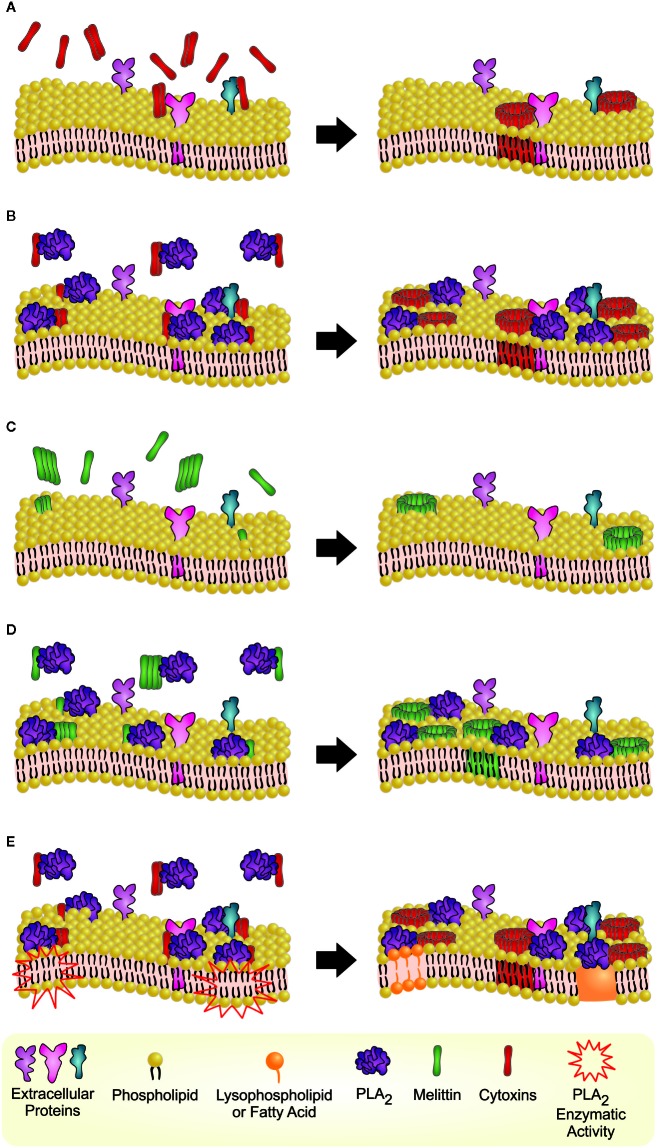
Hypothetical phenomena of synergistically enhanced cell lysis mediated by cytotoxin-PLA_2_ or melittin-PLA_2_ hetero-oligomers on phospholipid bilayer membranes. **(A)** Cytotoxins (monomers or oligomers) likely bind to anionic extracellular proteins or carbohydrates (Harvey, 2018), which may lead to further oligomerization and formation of membrane pores, leading to cell lysis (Forouhar et al., 2003; Konshina et al., 2011). **(B)** When combined with PLA_2_s, cytotoxins are more likely to easily bind to the neutral outer membrane on the lipid binding surface of the molecule (Singer et al., 2002; Burke and Dennis, 2009), resulting in synergistically enhanced lysis. **(C)** Different to cytotoxins, which are proteins of predominantly beta sheet structure, the helical structure of melittin provides better opportunities for melittin interaction with neutral membranes (Dempsey, 1990; Kleinschmidt et al., 1997), which is possibly mediated *via* electrostatic attraction between basic amino acid residues of melittin and the phosphate group of phosphatidylcholine (Dempsey, 1990; Kleinschmidt et al., 1997). The melittin-phospholipid binding enables melittin oligomerization, formation of membrane pores, and lysis (Yang et al., 2001; Pucca et al., 2019). **(D)** As for cytotoxins, PLA_2_s can also facilitate melittin binding in the cell membrane, resulting in synergistically enhanced lysis. **(E)** PLA_2_-induced hydrolysis also affects membrane integrity through the detergent action of the hydrolytic products of phospholipids, which may also contribute to the lytic effects (Lomonte and Gutiérrez, 2011). The last hypothetical phenomenon is unlikely to account for the synergistic effects observed for MII, since this toxin lacks enzymatic activity.

To investigate the possible occurrence of cytotoxin-PLA_2_ complexes, a pull-down assay was performed. The results indicated that formation of a complex took place between cytotoxins and PLA_2_s independent of the tested toxin combination (**Figure 3**). Thus, these data demonstrate that cytotoxins and PLA_2_s from the same and different species can form complexes, and that these complexes could be responsible for the synergistically enhanced cytotoxic effects observed on keratinocyte bilayer membranes. The hypothesized mechanism of hetero-oligomer complex interaction with membranes and complex-induced lytic effect is represented in **Figure 4**.

## Discussion

Toxin synergism between groups of toxins is a vast field of study that remains largely unexplored, since most toxin-toxin interactions are yet to be studied. However, cytotoxins, PLA_2_s, snake venom metalloproteinases (SVMPs), and snake venom serine proteases (SVSPs) are among the toxin families that possess the ability to interact synergistically with other toxins, which has been investigated in previous studies (Xiong and Huang, 2018). For instance, it has been demonstrated that a combination of acidic and basic svPLA_2_s (including Asp49 and Lys49 subtypes) can be found in a single snake species (Angulo and Lomonte, 2009), having synergistic effects (Mora-Obando et al., 2014; Bustillo et al., 2019). This is in agreement with the belief that toxin synergism can be identified whenever predominant protein families of snake venoms are co-administered (Xiong and Huang, 2018). In this relation, cobra snake venoms are known to be dominated by cytotoxins and PLA_2_s. In fact, around 95% of *N. nigricollis* and *N. mossambica* venom is composed of cytotoxins (72.8% and 67.7%, respectively) and PLA_2_s (21.9% and 27.1%, respectively) (Petras et al., 2011). Bee venom is also dominated by melittin (50%) and PLA_2_ (15%) toxins (Prado et al., 2010). Thus, due to the high abundance in venoms of cytotoxins and PLA_2_s, their interactions have been studied for decades and are considered great examples of protein complementation serving to potentiate biological activity (Gasanov et al., 2014). However, the mechanism behind this synergism phenomenon has not been elucidated.

Toxin synergism can be achieved through several mechanisms, mainly divided into intermolecular synergism, where toxins act on different targets or processes causing increased toxicity, or through supramolecular synergism, where toxins either interact with the same target or associate into a complex with increased toxicity (Laustsen, 2016). Gasanov et al. dedicated many efforts to propose a model for cytotoxin-PLA_2_ interaction (Gasanov et al., 1995; Gasanov et al., 1997; Gasanov et al., 2014). Moreover, methods like chromatography (gel filtration) (Mukherjee, 2010), ELISA (Saini et al., 1997), and functional assays (Louw and Visser, 1978; Chaim-Matyas et al., 1995) have been used successfully to prove that formation of non-covalent complexes between cytotoxins and PLA_2_ occurs. Here, we demonstrate that MII, a basic Lys49 PLA_2_ from *B. asper* with no enzymatic activity, produced a strong or very strong synergistic effect with different cytotoxins (Nn18, Nm17, Nn20, Nmo9) and melittin. The interaction of cytotoxins with this type of viper PLA_2_ has never been studied before. Our data with non-enzymatic MII are also supported by a recent study, which demonstrated that Asp49 and Lys49 PLA_2_s from *Bothrops diporus* venom present synergistic effects, and that the Lys49 variant (lacking enzymatic activity) has greater myotoxicity, cytotoxicity, anti-adhesion activity, and causes stronger inhibition of cell migration (Bustillo et al., 2019). In addition, to the best of our knowledge, it is the first time that the presence of supramolecular synergistic interaction between cytotoxins and PLA_2_ is demonstrated using a pull-down assay. In this study we demonstrate that independently of the animal species from which the tested venom toxin is derived, cytotoxins and PLA_2_s interacted and formed hetero-oligomers. Hence, we speculate that cytotoxin-PLA_2_ oligomers are generated in the venom gland and their cytotoxic effect is synergistically enhanced by this formation of hetero-oligomers. To support our hypothesis, a literature review was performed.

Due to their cationic nature, cytotoxins are likely to have no or very weak electrostatic interaction with phospholipids on the outer membrane leaflet of a mammalian cell, mostly composed of neutral components, unfavorable for interacting with the cytotoxins (Harvey, 1985). Since most of the negatively charged lipids are located in inner membrane leaflet (cytoplasmic face), cytotoxins likely first bind to anionic extracellular proteins or carbohydrates (e.g., oligosaccharides) (Harvey, 2018). Thus, cytotoxins (monomers or oligomers) seek and interact with unknown membrane anionic moieties through their basic electrostatic field. The cytotoxin–protein binding may enable cytotoxins to penetrate the membrane through their hydrophobic first loop and interact with negatively charged lipids from the inner membrane leaflet, which may lead to further oligomerization and formation of membrane pores, leading to cell lysis (Forouhar et al., 2003; Konshina et al., 2011). The cytotoxin-mediated mechanism of pore formation is like the perforin polymerization used by cytotoxic effector cells (Natural Killer cells and cytotoxic T lymphocytes) (Abbas et al., 2016). No specific protein targets have yet been identified for cytotoxins (Gasanov et al., 2014).

Different from cytotoxins, all three groups of PLA_2_s tested (IA, IIA, and III) bind readily to the neutral outer membrane through interactions with a group of hydrophobic residues on the lipid binding surface of the molecule (Singer et al., 2002; Burke and Dennis, 2009). Moreover, in case of MII, it was demonstrated that the toxin can also bind to fifteen different proteins, including nucleolin (Massimino et al., 2018). Thus, within the complex formation, PLA_2_s likely facilitate cytotoxin binding and penetration into the membrane, thereby enhancing the cytotoxin activity, which results in synergistically enhanced lysis. Supporting the synergistic activity, most PLA_2_s hydrolyze the ester bond of glycerophospholipids located at position two (*sn*-2), resulting in a structural change of the cell membrane and lysis. Although the composition of glycerophospholipids is diverse among mammalian cells and their distribution is different in the inner or outer plasma membrane leaflets (Hishikawa et al., 2014), PLA_2_-induced hydrolysis also affects membrane integrity through the detergent action of the hydrolytic products of phospholipids (i.e., lysophospholipids and fatty acids) contributing to the lytic effect as well (Lomonte and Gutiérrez, 2011). In addition, the augmentation of lysophospholipids facilitates flip-flopping of phospholipids and better exposure of acidic lipids to cytotoxins on the outer membrane leaflet (Gasanov et al., 1997).

Chaim-Matyas and co-authors have shown that among all different combinations of the P4 cytotoxin from *N. nigricollis* and PLA_2_s from different origins, the highest synergistic activity is seen between P4 and one of the basic PLA_2_s found in the same venom (Chaim-Matyas et al., 1995). Very strong synergism between intraspecies toxins was also observed in our study, where melittin and bvPLA_2_ combinations exhibited the highest cytotoxicity in N/TERT cells (>90% cytotoxicity and CTI <0.2). Interspecies toxin combinations were also tested, and interestingly, for snakes, we found that Nmo12, an acidic PLA_2_ from *N. mossambica*, exhibited less synergistic effects when combined with *Naja* spp. cytotoxins, compared to when it was combined with MII from the *B. asper* pit viper. Although more studies must be conducted for the evaluation of interfamily synergism, our results might indicate that synergism is not solely dependent on the toxins co-evolving within the same genus but may be a more universal feature co-evolving across genera and families. On the other hand, combinations employing cobra cytotoxins and bvPLA_2_ resulted in lower synergism compared to combinations with cobra cytotoxins and snake- derived PLA_2_s. This observation is not surprising, as it is to be expected that PLA_2_s and cytotoxins originating from snakes have co-evolved to result in the highest level of toxicity, just as the case is for PLA_2_s and melittin from bees. All in all, our data from 12 cross-species combinations indicate that synergistic effects of toxin-toxin combinations may be less dependent on species and more related to the fundamental structural and biochemical characteristics of the proteins themselves. Notably, in this study, not all toxins were isolated in high purity upon venom fractionation. Some of the cytotoxins and phospholipases were observed to have co-eluted in some fractions. However, synergistic effects were still observed when either fractionated or purified toxins were combined.

This is the first study that evaluates the cytotoxic effects of co-administration of cytotoxins and PLA_2_s on human keratinocytes. Many other cell types have been used for assessment of cytotoxic effects of venom cytotoxins and/or PLA_2_s, including erythrocytes, lymphocytes, cardiac myocytes, spleen cells, endothelial cells, skeletal muscle myoblasts/myotubes, and various tumor cells (Chaim-Matyas et al., 1991; Gasanov et al., 1997; Gasanov et al., 2014). However, to the best of our knowledge, there exists no studies reporting the effect of these individual toxins (or in combination) on human keratinocytes, which are among the most affected cells in cases of cobra bite-induced dermonecrosis (Rivel et al., 2016) or skin necrosis caused by bee venom (Palm and Medzhitov, 2013). The lack of studies using human keratinocyte cell lines could be justified by the limited availability of primary keratinocytes to generate epidermal models. Here, we demonstrate that N/TERT cells can be a biologically relevant target for *in vitro* studies with toxins (Smits et al., 2017).

## Conclusion and Final Remarks

This study demonstrates how cytotoxins and PLA_2_s from different species (elapids, vipers, and bees) may interact synergistically to enhance cell lysis, explored via the use of a human keratinocyte assay mimicking human skin *in vitro*. The results indicate that strong to very strong synergism may result from the hetero-oligomerization of cytotoxins and PLA_2_s to potentiated toxin complexes, which are speculated to be better posed to interact with and disrupt cellular membranes. Finally, based on the results obtained in this work combined with findings extracted from prior art, we propose a mechanistic model for how cytotoxin and PLA_2_ complex formation may possibly mediate synergistically enhanced cell lysis. We further demonstrate that toxin synergism between cytotoxins, cytotoxin-like toxins, and PLA_2_s may occur across snake genera, snake families, and even entirely different species (snakes and bees) due to the fundamental structural and biochemical characteristics of the toxins themselves.

## Data Availability Statement

All datasets generated for this study are included in the article/**Supplementary Material**.

## Author Contributions

MP, SA, FÇ, TS, and ES performed experiments and analyzed results. UK and FM provided the cell line and FM helped with the cell culture experiments. SA, CS, and BL purified and biotinylated the toxins. FAC performed the figure illustration. MP, SA, LL, and AL wrote the paper. BL, UK, EA, and FÇ gave their valuable and professional suggestions. All authors corrected the manuscript and provided revisions.

## Funding

We thank Conselho Nacional de Desenvolvimento Científico e Tecnológico (CNPq, The National Council for Scientific and Technological Development, grant no. 307155/2017-0); Fundação de Amparo à Pesquisa do Estado de São Paulo (FAPESP, São Paulo Research Foundation, grant no. 2017/04724-4, scholarship to FAC no. 2017/14035-1 and 2018/14158-9), and the Villum Foundation (grant 00025302).

## Conflict of Interest

The authors declare that the research was conducted in the absence of any commercial or financial relationships that could be construed as a potential conflict of interest.

## Supplementary Material

Supplementary Table 1 Toxin purities. Snake cytotoxin (CTx) and phospholipase A_2_ (PLA_2_) purities were evaluated using *De Novo* sequencing. The abundance results received were filtered to exclude results with low reliability. Results with less than two peptide matches and lower than 30% sequence coverage were removed from the dataset. In order to obtain purities, the abundance results for each fraction were summed and divided by each respective protein that matched the peptide sequences from the screen. The cytotoxin and phospholipase A_2_ purities from each fraction were grouped to determine the purities relative to their toxin families. The Uniprot IDs listed made up the primary abundances for each fraction when screened from the Uniprot database during *De Novo* sequencing. Melittin and honeybee phospholipase A_2_ (bvPLA_2_) were purchased from Sigma-Aldrich and are displayed with their listed purities.

Supplementary Figure 1 Electrophoretic profiles of toxins. Toxins (Nn18, Nn20, Nn25, Nn17, Nmo9, Nmo12, melittin, Bee-PLA2, and M-II – 2 µg) were evaluated using Tris-Tricine SDS-PAGE 16% under reducing conditions using Coomassie blue staining.
