# Supplementary material for: Unity Makes Strength: Exploring Intraspecies and Interspecies Toxin Synergism between Phospholipases A2 and Cytotoxins
Source: Front Pharmacol. 2020 May 7;11:611. doi: 10.3389/fphar.2020.00611 (PMC7221120; doi:10.3389/fphar.2020.00611)
Supplement: Supplementary Table 1 — Toxin purities. Snake cytotoxin (CTx) and phospholipase A2 (PLA2) purities were evaluated using De Novo sequencing. The abundance results received were filtered to exclude results with low reliability. Results with less than two peptide matches and lower than 30% sequence coverage were removed from the dataset. In order to obtain purities, the abundance results for each fraction were summed and divided by each respective protein that matched the peptide sequences from the screen. The cytotoxin and phospholipase A2 purities from each fraction were grouped to determine the purities relative to their toxin families. The Uniprot IDs listed made up the primary abundances for each fraction when screened from the Uniprot database during De Novo sequencing. Melittin and honeybee phospholipase A2 (bvPLA2) were purchased from Sigma-Aldrich and are displayed with their listed purities. [file Table_1.pdf]

| Fraction (toxin): | Uniprot ID: | Purity %: |
|---|---|---|
| Nmo9 (CTx) | ~ P01468 | 41,1 |
| Nm17 (CTx) | ~ P01474 | 84,4 |
| Nn18 (CTx) | ~ P01468 | 93,4 |
| Nn20 (CTx) | ~ P01468 | 63,0 |
| Nmo12 ($PLA_2$) | ~ P00605 | 35,5 |
| Melittin | P01501 | 97,0 |
| Bv$PLA_2$ | B7UUK1 | 85,0 |
| Myotoxin II | ~ P24605 | 100,0 |
