# Supplementary figures and images for: Unity Makes Strength: Exploring Intraspecies and Interspecies Toxin Synergism between Phospholipases A2 and Cytotoxins
Source: Front Pharmacol. 2020 May 7;11:611. doi: 10.3389/fphar.2020.00611 (PMC7221120; doi:10.3389/fphar.2020.00611)

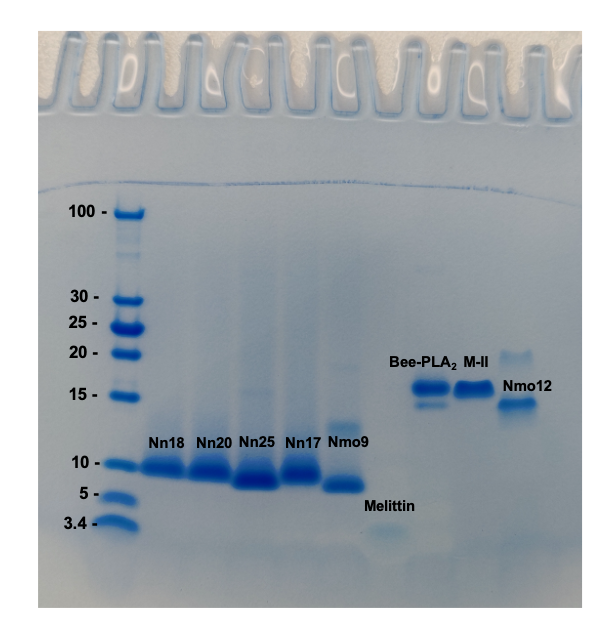

Supplement: Supplementary Figure 1 — Electrophoretic profiles of toxins. Toxins (Nn18, Nn20, Nn25, Nn17, Nmo9, Nmo12, melittin, Bee-PLA2, and M-II – 2 µg) were evaluated using Tris-Tricine SDS-PAGE 16% under reducing conditions using Coomassie blue staining. [file Image_1.png]
